# Antimicrobial activity of essential oils and carvacrol, and synergy of carvacrol and erythromycin, against clinical, erythromycin-resistant Group A Streptococci

**DOI:** 10.3389/fmicb.2015.00165

**Published:** 2015-03-03

**Authors:** Gloria Magi, Emanuela Marini, Bruna Facinelli

**Affiliations:** Unit of Microbiology, Department of Biomedical Sciences and Public Health, Polytechnic University of Marche, AnconaItaly

**Keywords:** group A streptococci, essential oils, thyme, origanum, carvacrol, erythromycin, synergy

## Abstract

In the present study, we have evaluated the *in vitro* antibacterial activity of essential oils from *Origanum vulgare, Thymus vulgaris, Lavandula angustifolia, Mentha piperita,* and *Melaleuca alternifolia* against 32 erythromycin-resistant [Mininum Inhibitory Concentration (MIC) ≥1 μg/mL; inducible, constitutive, and eﬄux-mediated resistance phenotype; *erm*(TR), *erm*(B), and *mef*(A) genes] and cell-invasive Group A streptococci (GAS) isolated from children with pharyngotonsillitis in Italy. Over the past decades erythromycin resistance in GAS has emerged in several countries; strains combining erythromycin resistance and cell invasiveness may escape β-lactams because of intracellular location and macrolides because of resistance, resulting in difficulty of eradication and recurrent pharyngitis. Thyme and origanum essential oils demonstrated the highest antimicrobial activity with MICs ranging from 256 to 512 μg/mL. The phenolic monoterpene carvacrol [2-Methyl-5-(1-methylethyl) phenol] is a major component of the essential oils of *Origanum* and *Thymus* plants. MICs of carvacrol ranged from 64 to 256 μg/mL. In the live/dead assay several dead cells were detected as early as 1 h after incubation with carvacrol at the MIC. In single-step resistance selection studies no resistant mutants were obtained. A synergistic action of carvacrol and erythromycin was detected by the checkerboard assay and calculation of the Fractional Inhibitory Concentration (FIC) Index. A 2- to 2048-fold reduction of the erythromycin MIC was documented in checkerboard assays. Synergy (FIC Index ≤0.5) was found in 21/32 strains and was highly significant (*p* < 0.01) in strains where resistance is expressed only in presence of erythromycin. Synergy was confirmed in 17/23 strains using 24-h time-kill curves in presence of carvacrol and erythromycin. Our findings demonstrated that carvacrol acts either alone or in combination with erythromycin against erythromycin-resistant GAS and could potentially serve as a novel therapeutic tool.

## INTRODUCTION

The increase in antibiotic-resistant bacteria has revived the interest in plant products as alternative/adjunct antimicrobial agents to control pathogenic micro-organisms (Cowan, 1999; Hemaiswarya et al., 2008; Hyldgaard et al., 2012). A major group of plant antimicrobial compounds is represented by essential oils, which are complex mixtures of volatile secondary metabolites. They are used in the food industry because of their preservative potency against food-borne pathogens—thanks to their antimicrobial, antibacterial, and antifungal properties. Besides antimicrobial activity, essential oils and their components can act in synergy with some antibiotics, enhancing their antimicrobial activity (Langeveld et al., 2014). The phenolic monoterpene carvacrol [2-Methyl-5-(1-methylethyl) phenol, isomeric with thymol] is a major component of the essential oils of plants of the Labiatae family, including *Origanum* and *Thymus*, which are commonly used as seasoning and in traditional medicine since ancient times (Nostro and Papalia, 2012). Carvacrol has been classified as GRAS (Generally Recognized As Safe) and approved for food use (EAFUS, 2006; Hyldgaard et al., 2012; European Parliament and Council, 1996). Beside anti-inflammatory, antioxidant, antitumor, analgesic, anti-hepatotoxic, and insecticidal properties, several studies have demonstrated that carvacrol has antimicrobial properties (Hyldgaard et al., 2012). Findings regarding the latter properties are the subject of a recent review (Hyldgaard et al., 2012). The antibacterial action of carvacrol, which is stronger against Gram-positive than Gram-negative bacteria, principally relies on bacterial membrane damage; it results in dissolution of the proton motive force and subsequent reduction in ATP synthesis that lead to reduction in other energy-dependent cell processes, including synthesis of enzymes and toxins (Nostro and Papalia, 2012). In particular, carvacrol has extensively been tested as an antimicrobial agent in food to control Gram-positive and Gram-negative pathogens, including *Bacillus cereus*, *Enterococcus faecalis*, *Listeria monocytogenes*, *Staphylococcus aureus*, *Escherichia coli* O157:H7, *Pseudomonas fluorescens*, *Salmonella typhimurium*, *Vibrio cholerae*, and *V. vulnificus* (Hyldgaard et al., 2012; Langeveld et al., 2014). The ability of carvacrol to exert synergistic effects in combination with a number of antibiotics, including macrolides, has been recently reported (Langeveld et al., 2014).

Erythromycin and related molecules are the second-line drugs used to treat pharyngotonsillitis and other infections caused by *Streptococcus pyogenes* [Group A streptococci (GAS)], an important human pathogen. GAS cause a variety of clinical manifestations ranging from non-invasive disease, such as pharyngitis and impetigo, to more severe, invasive infections including necrotizing fasciitis, sepsis, toxic shock-like syndrome, and post-streptococcal *sequelae* such as acute rheumatic fever, rheumatic heart disease, and glomerulonephritis (Cunningham, 2008). In particular, GAS are the most common bacterial cause of pharyngotonsillitis in children. Over the past decades erythromycin resistance in GAS has emerged in several countries, including Italy (Kaplan and Cornaglia, 2005; Gracia et al., 2009). At present, it has fallen dramatically in Europe and America, while rates >90% have recently been reported in China (Chang et al., 2010; Liang et al., 2012). Several genes are responsible for erythromycin resistance in GAS (Giovanetti et al., 2002). An eﬄux mechanism encoded by different macrolide eﬄux (*mef*) genes causes resistance to 14- and 15-membered macrolides (M phenotype); in contrast, ribosomal modification by erythromycin resistance methylase (*erm*) genes causes co-resistance to macrolide-lincosamide-streptogramin B (MLS) antibiotics. MLS resistance is constitutive when it is encoded by the *erm*(B) gene (cMLS phenotype); it is inducible, i.e., expressed only in presence of the antibiotic, when it is encoded by the *erm*(TR) or the variant *erm*(B) gene (iMLS phenotype). An association between erythromycin resistance and ability to invade and survive within human respiratory cells has been documented in Italy among GAS isolated from children with pharyngotonsillitis (Facinelli et al., 2001): these strains may escape β-lactams because of intracellular location and macrolides—which unlike β-lactams enter eukaryotic cells and are active in intracellular compartments—because of resistance, resulting in difficulty of eradication and recurrent pharyngitis.

In this study, we evaluated the antibacterial activity of different essential oils and of carvacrol, alone and in combination with erythromycin, against erythromycin-resistant GAS isolated from children with pharyngotonsillitis in Italy.

## MATERIALS AND METHODS

### GAS STRAINS AND GROWTH MEDIA

Test strains were 32 erythromycin-resistant [Mininum Inhibitory Concentration (MIC) ≥1 μg/mL] GAS strains isolated from children with pharyngotonsillitis in a nationwide survey in Italy (Varaldo et al., 1999; **Table 1**). Erythromycin-resistant GAS isolates were genotypically and phenotypically heterogeneous: *erm*(TR)/iMLS (*n* = 6); *erm*(B)/iMLS (*n* = 6); *erm*(B)/cMLS (*n* = 8); *mef*(A)/M (*n* = 12). The strains had previously been investigated for the association between erythromycin resistance and ability to enter and persist inside human respiratory A549 cells (Facinelli et al., 2001; Spinaci et al., 2004, 2006); they had also been typed at the molecular level (Spinaci et al., 2004, 2006). Each of the 32 strains included in the study represents a clone identified among Italian erythromycin-resistant GAS. Blood agar base (BAB) supplemented with 5% sheep blood, Müller-Hinton agar (MHA) supplemented with 5% sheep blood, Müller-Hinton cation-adjusted broth (CAMHB) supplemented with 3% laked sheep blood, and brain heart infusion broth (BHIB; Oxoid, Basingstoke, UK) were used throughout the study. Isolates were maintained in glycerol at -70^∘^C and sub-cultured twice on BAB before testing.

**Table 1:** Characteristics of erythromycin-resistant GAS, each strain representing an erythromycin-resistant Italian GAS clone.

Genotype/phenotype of erythromycin resistance (no. of strains)	Mininum Inhibitory Concentration (MIC; μg/mL)				Fractional Inhibitory Concentration (FIC) Index			
	ERY^a^	CAR^b^	Best combination^c^		FIC		≤0.5	>0.5
			ERY^a^	CAR^b^	ERY^a^	CAR^b^		
***erm*(TR)/iMLS (*n* = 6)**
SP1900^d^	256	128	4	32	0.0156	0.2500	0.2656	
SP1161	8	64	1	8	0.1250	0.1250	0.2500	
SP55	8	128	0.5	32	0.0625	0.2500	0.3125	
SP1160	16	128	4	8	0.2500	0.0625	0.3125	
SP4502	64	128	16	8	0.2500	0.0625	0.3125	
11613	8	64	2	16	0.2500	0.2500	0.5000	
***erm*(B)/iMLS (*n* = 6)**
SP1188	512	128	0.5	32	0.0009	0.2500	0.2509	
11414	512	128	0.5	32	0.0009	0.2500	0.2509	
11107	512	128	1	32	0.0019	0.2500	0.2519	
SP1181	512	128	2	32	0.0039	0.2500	0.2539	
SP114	512	128	0.5	32	0.0009	0.2500	0.2509	
SP1189	512	64	0.25	32	0.0005	0.5000		0.5005
***erm*(B)/cMLS (*n* = 8)**
SP2130	512	128	2	32	0.0039	0.2500	0.2539	
10039	512	128	2	32	0.0039	0.2500	0.2539	
SP9707	512	64	0.25	32	0.0005	0.5000		0.5005
SP9103	512	64	0.25	32	0.0005	0.5000		0.5005
37014	512	64	0.25	32	0.0005	0.5000		0.5005
SP1791	512	64	0.25	32	0.0005	0.5000		0.5005
SP1003	512	128	0.25	64	0.0005	0.5000		0.5005
SP1070	512	128	0.25	64	0.0005	0.5000		0.5005
***mef*(A)/M (*n* = 12)**
10911	8	64	2	16	0.2500	0.2500	0.5000	
SP9721	16	64	0.5	16	0.0313	0.2500	0.2813	
9407	32	64	8	16	0.2500	0.2500	0.5000	
SP1180	16	128	4	32	0.2500	0.2500	0.5000	
10239	16	128	4	32	0.2500	0.2500	0.5000	
SP118	16	256	0.5	64	0.0313	0.2500	0.2813	
10244	16	128	4	32	0.2500	0.2500	0.5000	
10313	16	128	4	32	0.2500	0.2500	0.5000	
SP1013	1	128	0.25	64	0.2500	0.5000		0.7500
13962	16	128	0.5	64	0.0313	0.5000		0.5313
128–192	16	128	0.5	64	0.0313	0.5000		0.5313
SP1951	16	128	0.03	64	0.0019	0.5000		0.5019

^a^Erythromycin; ^b^Carvacrol; ^c^Combination of carvacrol and erythromycin giving thelowest FIC Index value; ^d^(Palmieri et al., 2006).

### ANTIMICROBIALS AND SUSCEPTIBILITY TESTS

Five different essential oils [origanum (W282812), thyme (W306401), lavender (W262218), tea tree (W390208), and peppermint (W284815)], carvacrol (W224502, >98% purity), and erythromycin (E-5389) were all purchased from Sigma–Aldrich (St. Louis, MO, USA). Essential oils (10 mg/mL stock solutions) were stored in DMSO at -20^∘^C; carvacrol and erythromycin (10 mg/mL stock solutions) were stored in absolute ethanol at -20^∘^C. MIC is defined as the lowest concentration of an antimicrobial that will inhibit the visible growth of a microorganism after overnight incubation. MICs of essential oils and carvacrol were determined by agar dilution (in blood-supplemented MHA) and microdilution (in blood-supplemented CAMHB) methods, respectively, as recommended by Clinical and Laboratory Standards Institute [CLSI] (2014) guidelines. All experiments were performed in triplicate.

### LIVE/DEAD ASSAY

Group A streptococci survival in presence of carvacrol was studied by the live/dead assay as described previously (Zandri et al., 2012) using SYBR Green I (Invitrogen, Eugene, OR, USA) and propidium iodide (Sigma–Aldrich), two nucleic acid dyes differing in their ability to penetrate bacterial cells: bacteria with intact cell membranes stain fluorescent green, whereas those with damaged membranes stain fluorescent red. Briefly, overnight grown streptococci were suspended in 1 mL carvacrol-supplemented BHIB (∼1 × 10^8^ CFU/mL) and incubated for 1, 3, or 24 h at 37^∘^C in 5% CO_2_. After staining with 1 × SYBR Green I and 40 μg/mL propidium iodide, samples were incubated at room temperature for 25 min in the dark, harvested on GTBP filters (Ø = 0.2 μm, Millipore, Billerica, MA, USA), and examined under an epifluorescence microscope (Axioskop 2, Zeiss, Milano, Italy).

### CHECKERBOARD TEST

Synergy was tested by the checkerboard method, a two-dimensional array of serial concentrations of test compounds, that has been used most frequently to assess antimicrobial combinations *in vitro* (Pillai et al., 2005). The tested dilutions were based on the MIC of the two substances. The checkerboard test was used as the basis to calculate a Fractional Inhibitory Concentration (FIC) Index (Pillai et al., 2005) according to the formulas: FIC_A_ = MIC_A+B_/MIC_A_, FIC_B_ = MIC_B+A_/MIC_B_, FIC Index = FIC_A_+FIC_B_. The MIC_A+B_ value is the MIC of compound A in the presence of compound B, and *vice versa* for MIC_B+A_. FIC Index values were interpreted accordingly to Odds (Odds, 2003): synergy (FIC Index ≤0.5), antagonism (FIC Index >4.0), and no interaction (FIC Index >0.5–4.0).

The test was performed in blood-supplemented CAMHB using 96-well microtiter plates containing erythromycin and carvacrol in twofold serial concentrations. Bacterial suspensions were prepared to yield final inocula of ∼5 × 10^5^ CFU/mL. Plates were read after overnight incubation at 37^∘^C in 5% CO_2_. Each test was performed in triplicate. Test results were also represented by isobolograms constructed by plotting synergistic concentrations of carvacrol and erythromycin (Mulyaningsih et al., 2010).

### TIME-KILL CURVES

Time-kill experiments were performed in BHIB in microtiter plates containing different combinations of carvacrol and erythromycin at different sub-MICs. Briefly, streptococci (∼5 × 10^5^ CFU/mL) were placed on microtiter plates, incubated for 24 h at 37^∘^C and read at OD_690_ at 1-h intervals using Multiscan Ascent (Thermo Scientific, Waltham, MA, USA). Controls also included growth in presence of carvacrol and erythromycin alone. All experiments were performed in triplicate.

### SEARCH FOR CARVACROL MUTANTS

In these experiments, bacterial cells were grown overnight in BHI agar plates, scraped off, washed once with BHIB, and resuspended to a final concentration of 1 × 10^10^ to 1 × 10^11^ CFU/mL. An aliquot (50 μL) of bacterial suspension was spread on carvacrol-containing BAB plates at 1, 2, and 4 times the MIC. The plates were incubated at 37^∘^C in CO_2_ for 72 h. Experiments were repeated twice.

### STATISTICAL ANALYSIS

Synergy data were analyzed using Fisher’s exact test. Fisher’s test was used to evaluate whether the association between phenotype iMLS and synergy in *erm*-carrying strains was significant. Significance was set at *p*-value < 0.01.

## RESULTS

**FIGURE 1:**
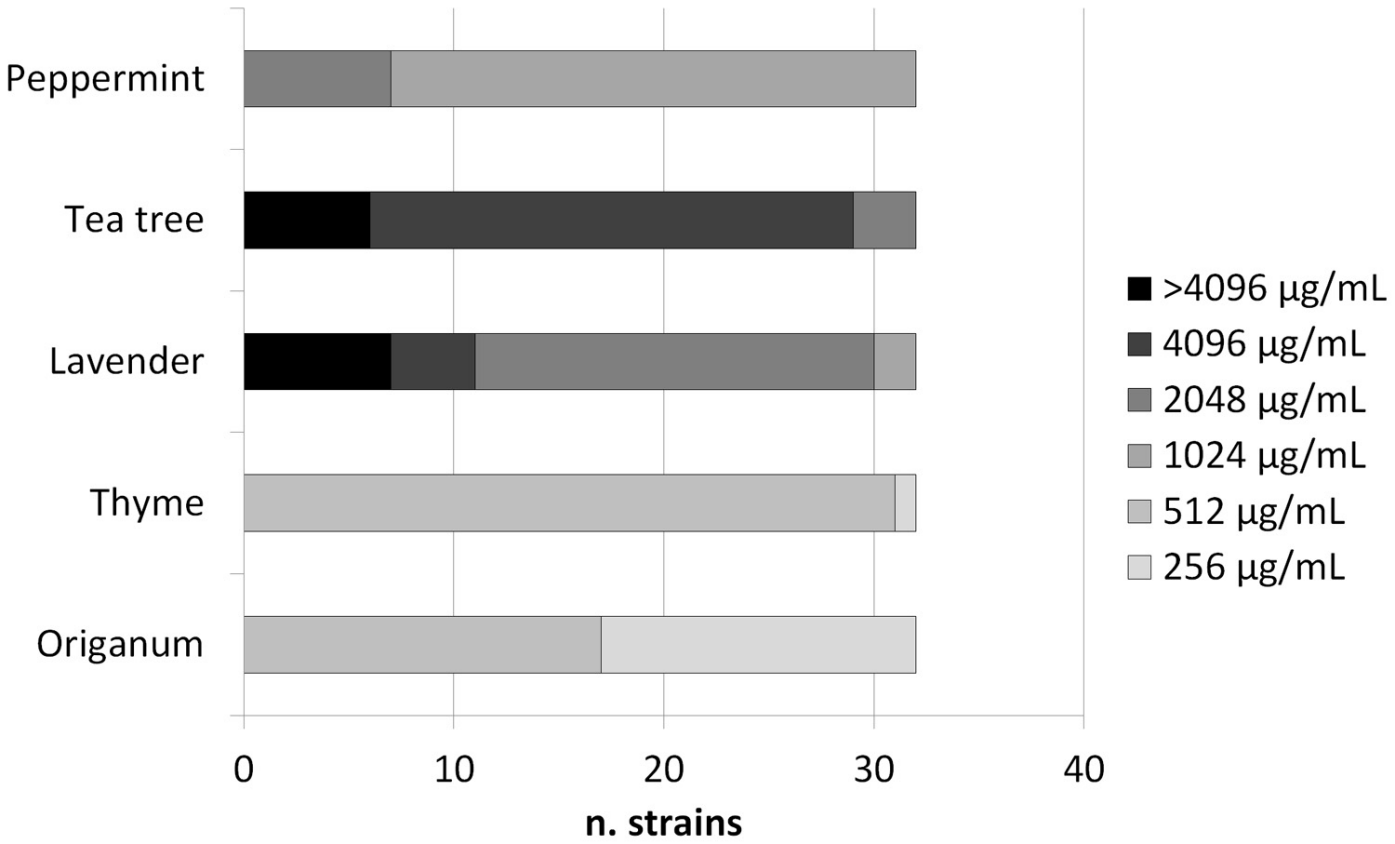
**Mininum Inhibitory Concentrations (MICs) of five essential oils against GAS strains**.

The MICs of origanum and thyme oils ranged from 256 to 512 μg/mL, while those of lavender, tea tree, and peppermint oils ranged from 1024 to >4096 μg/mL (**Figure 1**). The MICs of carvacrol were 64 μg/mL (*n* = 10 strains), 128 μg/mL (*n* = 21 strains), and 256 μg/mL (*n* = 1 strain; **Table 1**). In the live/dead assay several red cells were detected as early as 1 h after incubation with carvacrol at the MIC (data not shown).

**FIGURE 2:**
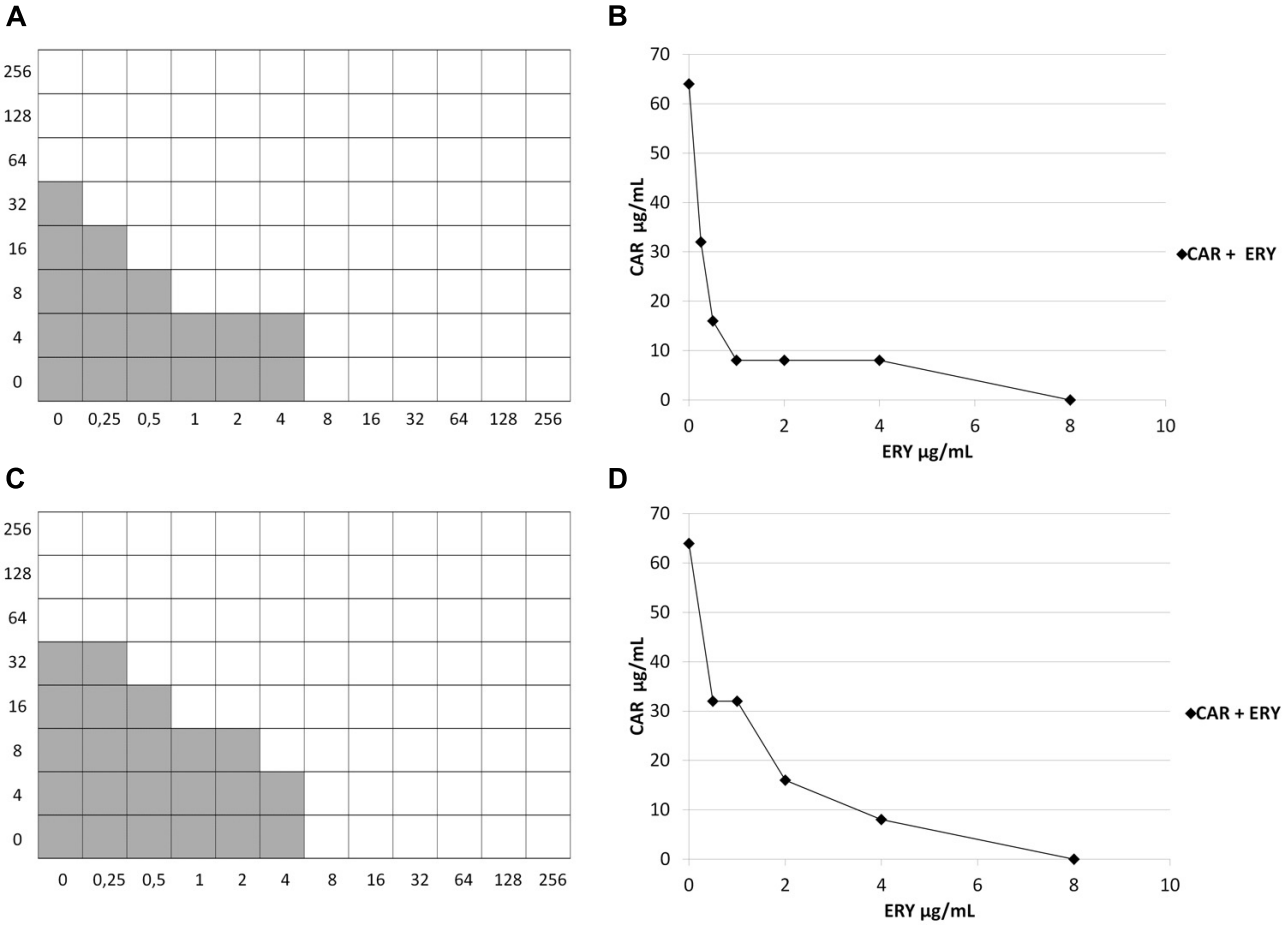
**Checkerboard test and isobolograms. (A,B)** strain SP1161 [Fractional Inhibitory Concentration (FIC) Index = 0.2500]; **(C,D)** strain 10911 (FIC Index = 0.5000). Shading, visible growth. CAR, carvacrol; ERY, erythromycin.

In the checkerboard assay, 77 different combinations of erythromycin and carvacrol were tested for each strain, ranging from several dilutions below the MIC to twice the MIC. A 2- to 2048-fold reduction of the erythromycin MIC was documented in synergy tests with the checkerboard assay (**Table 1**). FIC Index values were calculated by considering all combinations of carvacrol and erythromycin in which there was no visible growth. The “best combination” of carvacrol and erythromycin, i.e., the one giving the lowest FIC Index value, was indicated in **Table 1**. FIC Index values ranged from 0.25 to 0.75 (**Table 1**). Synergy (FIC Index ≤0.5) was detected in 21/32 strains (65.6%) and no interaction (FIC Index >0.5) in the remaining 11 strains (34.4%); antagonism was never observed. Strains where synergy was detected included all 6 *erm*(TR)/iMLS, 5/6 *erm*(B)/iMLS, 2/8 *erm*(B)/cMLS, and 8/12 *mef*(A)/M isolates. Among *erm*-carrying strains the association between the iMLS phenotype and synergy was very statistically significant (*p* = 0.0044). Strains where no interaction was detected included 8 strains with borderline FIC Index values (0.5005, 0.5019). Isobolographic analysis confirmed a synergistic effect against strains with FIC Index ≤0.5 (**Figure 2**) and no interaction against those with FIC Index >0.5, including those with borderline FIC Index values (data not shown).

**FIGURE 3:**
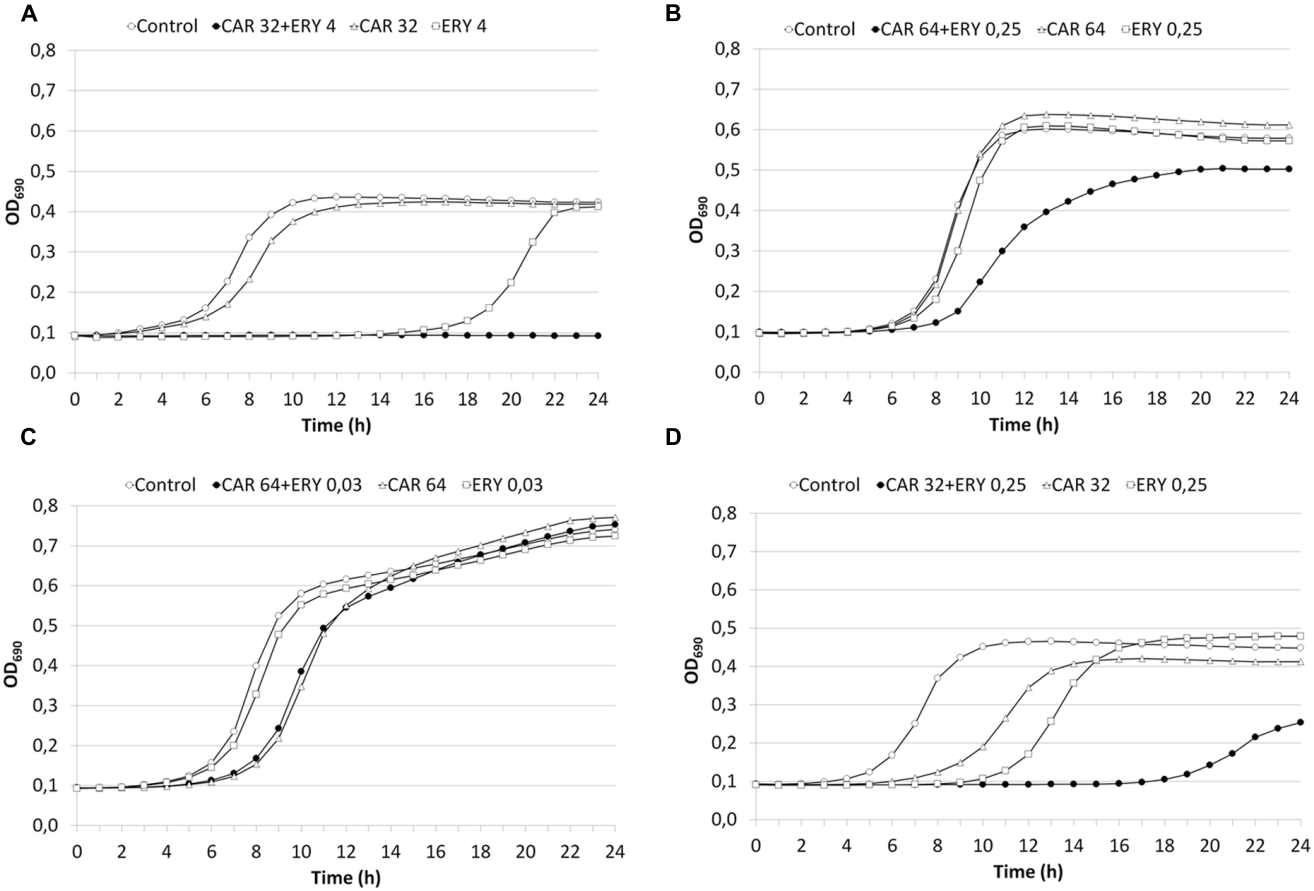
**Time-kill curves. (A)** strain SP1900 (FIC Index = 0.2656). **(B)** SP1013 (FIC Index = 0.7500). **(C)** SP1951 (FIC Index = 0.5019). **(D)** SP1189 (FIC Index = 0.5005). CAR, carvacrol; ERY, erythromycin.

Synergy was also examined in 23 strains (18 strains with FIC Index ≤0.5 and 5 strains with FIC Index >0.5) using 24-h time-kill curves in presence of carvacrol and erythromycin. These findings confirmed the checkerboard data in 13/18 strains with FIC Index ≤0.5 (8/9 iMLS, 1/1 cMLS, and 4/8 M; **Figure 3A**), and in 4/5 strains with FIC Index >0.5 (1 cMLS, and 3 M; **Figure 3B**). The time-kill curves of the two strains with borderline FIC Index values (SP1951, FIC Index 0.5019; and SP1189, FIC Index 0.5005) indicated a lack of interaction (**Figures 3C,D**), even though synergy was seen in strain SP1189 in the first 18 h (**Figure 3D**). The discrepancy between checkerboard and time-kill data may depend on the media (with/without blood) and/or growth conditions (with/without CO_2_) (Odds, 2003).

No carvacrol-resistant mutants were obtained in single-step resistance selection experiments using strains SP2130, SP1188, SP1900, and 10239.

## DISCUSSION

One of the alternative strategies to fight antibiotic-resistant bacteria is the use of natural antimicrobial substances such as plant essential oils and their components. Another possibility is to combine existing antibiotics with phytochemicals to enhance the efficacy of antibiotics. Among others, essential oils from *Thymus vulgaris* and *Origanum vulgare* contain carvacrol and thymol, whose antibacterial activity and synergistic effect in combination with antibiotics against food-related bacteria have been demonstrated (Hyldgaard et al., 2012; Langeveld et al., 2014).

In this study, the antimicrobial activity of five essential oils from different plants was tested against pharyngeal GAS isolates, all erythromycin-resistant and cell-invasive strains. The antimicrobial activity of thyme and origanum oils was high, with MICs much lower than those of lavender, mint, and tea tree oils. Since carvacrol is a major component of thyme and origanum essential oils, we decided to test the susceptibility of GAS strains to carvacrol. Our findings demonstrated a high activity of carvacrol against GAS, with MICs lower than those reported for other Gram-positive bacteria (Hyldgaard et al., 2012). A bactericidal action of carvacrol, probably through membrane damage, was suggested by the live/dead assay. Attempts to select carvacrol-resistant mutants were unsuccessful, suggesting that carvacrol is not prone to the development of resistance in GAS.

To our knowledge the synergistic activity of carvacrol and antibiotics against GAS has never been investigated, with the exception of a recent study of a single *erm*(B) collection strain (Palaniappan and Holley, 2010). Carvacrol in combination with erythromycin showed a synergistic action against tested GAS isolates; antagonism was never observed.

Interestingly, synergy was highly significant (*p* < 0.01) in iMLS strains, in which erythromycin resistance is only expressed in presence of an inducing sub-inhibitory concentration of the antibiotic. The molecular basis of the synergy deserves further investigation; an interference with the expression of the inducible methyl-transferases gene or inhibition of the enzyme itself might be involved in carvacrol’s synergistic action against iMLS GAS strains.

Overall, results of the current study suggest a potential use of origanum and thyme essential oils and of carvacrol against GAS. Moreover, the evidence of a synergism between erythromycin and carvacrol suggest a possible re-use of erythromycin against erythromycin-resistant GAS. In particular, carvacrol might be useful against those GAS strains which combine erythromycin resistance and the ability to invade and survive within human respiratory cells (Facinelli et al., 2001), resulting in difficulty of eradication and recurrent pharyngitis. With more knowledge of the mechanism underlying the synergism it may be possible to develop safe drug combinations.

## Conflict of Interest Statement

The authors declare that the research was conducted in the absence of any commercial or financial relationships that could be construed as a potential conflict of interest.
